# Post-traumatic pseudoaneurysm of the right hepatic artery: Two case reports – New Ukrainian reality

**DOI:** 10.1016/j.ijscr.2024.110143

**Published:** 2024-08-13

**Authors:** Eduard V. Svitlychnyi, Olha A. Kochmaruk

**Affiliations:** a Department of Military Surgery, Ukrainian Military Medical Academy, 01015, Kniaziv Ostrozkykh str. 45/1, bldg. 33, Kyiv, Ukraine; b Department of Ultrasound Diagnostics, Military Medical Department, SSU, 01601, Volodymyrska str. 33, Kyiv, Ukraine

**Keywords:** Pseudoaneurysm, Case series, Right hepatic artery, Ultrasound observation, Abdominal trauma, Surgery

## Abstract

**Introduction and significance:**

The diagnostics and treatment management in conditions of massive sanitary losses with the use of staged treatment have their own specifics and require a multidisciplinary approach with the involvement of a wide range of specialists and the use of modern technologies. The number of sources covering the ultrasound diagnostics and clinical course of hepatic artery pseudoaneurysm as a complication of gunshot wounds is quite limited in world literature.

**Case presentation:**

We present the experience of the observation and management of the right hepatic artery pseudoaneurysm in case of the blast injury of liver in two patients: the example of successful resolution with spontaneous occlusion and the example with the occurrence of internal bleeding as a result of pseudoaneurysm rupture.

**Clinical discussion:**

Clinical cases presented here belong to the category of severe injuries caused by high-energy weapons, which are characterized by a syndrome of mutual aggravation and need for simultaneous treatment of several damaged organs. The use of contrast methods in severely injured patients requires instrumental justification, and results of daily ultrasound monitoring with the use of color Doppler program can be the one.

**Conclusion:**

Pseudoaneurysm of hepatic arteries is a dangerous complication of severe liver wounds and injuries, which occurs in 3·2 % of patients according to our data. The method of ultrasound examination with the use of color Doppler mapping program allows to visualize pseudoaneurysms and monitor their progress. When identifying patients with pseudoaneurysm of hepatic arteries at the level II–III medical care (Role II–III), their further evacuation should be carried out to medical institutions equipped with endovascular correction technologies.

## Highlights

• Pseudoaneurysm of hepatic arteries is a dangerous complication of severe liver wounds and injuries
• Cases of the spontaneous thrombosis and cases of pseudoaneurysm rupture with a massive bleeding are seen
• Sonography method with the use of color Doppler mapping allows to visualize pseudoaneurysms and monitor their progress.
• Patients with pseudoaneurysm of hepatic arteries should be evacuated to medical institutions with endovascular technologies.

## 1 Introduction

Liver injuries as a result of combat traumas occur in 20–24 % of cases [[1], [2], [3]]. Significant damaging of the liver parenchyma with vascular and biliary duct lesions (Moore III–ІV, WSES III–IV) is characterized by the massive bleeding that doesn't stop on its own, and a bile leakage, and is often combined with other parenchymal and hollow organs injuries of abdomen and chest. Up to 75 % of wounded military servicemen are hospitalized in severe or extremely severe conditions. These factors determine favorable conditions for the occurrence of complications [9,11,16]. Stabilization of the patients is achieved only by repeated surgeries in accordance with DCS [21] (“Damage Control Surgery”) method. With liver injuries, one of the rare complications is the formation of a pseudoaneurysm of hepatic artery branches, which carries the risk of further rupture and a threat to a patient's life [4,5,13].

The diagnostics and treatment management in conditions of massive sanitary losses with the use of staged treatment have their own specifics and require a multidisciplinary approach with the involvement of a wide range of specialists and the use of modern technologies. The number of sources covering the ultrasound diagnostics and clinical course of hepatic artery pseudoaneurysm as a complication of gunshot wounds is quite limited in world literature.

Pseudoaneurysm of hepatic artery is a rupture of all blood vessel wall layers as a result of a severe trauma or injury with formation of a pulsating cavity without hemorrhage development due to a paravasal connective tissues “capsule” formation. With liver damage, it usually occurs in 0·4 %–4 % of injured and traumatized patients, has no specific manifestation until the moment of hemorrhage, and is most commonly diagnosed during an ultrasound examination [[6], [7], [8]]. The factors of organ lesion are the closed trauma as a result of blast wave impact, mechanical impact of a projectile fragmentation or a bullet with the wound channel formation, and a temporary pulsating cavity formed behind it, causing contusion and ruptures of the adjacent parenchyma [1,2]. And the search for the best diagnostic and treatment options is ongoing [[10], [11], [12], [13], [14], [15], [16], [17], [18]].

We present the experience of the observation and management of the right hepatic artery pseudoaneurysm in case of the blast injury of liver in two patients: the example of successful resolution with spontaneous occlusion and the example with the occurrence of internal bleeding as a result of pseudoaneurysm rupture.

There were 93 patients with liver injury under our observation at the level IV medical care (analogue of triage Role IV). Severe liver damage (Moore III–IV) occurred in 33 (35·5 %) cases, with 3 (3·2 %) cases of post-traumatic right hepatic artery pseudoaneurysms among them.

## 2 Case presentation

### 2.1 Clinical case #1

A 32-year-old military serviceman suffered a severe right-sided mine-blast thoraco-abdominal trauma with the lesion of the right diaphragm dome, the right kidney, a through hole liver injury (S5, S6, S7) with a parenchymal defect (Moore IV), colon injury, spleen rupture and right-sided hemopneumothorax. Medicines and medical devices regulated by the list of medications and devices prescribed for a certain pathology at a certain level of medical care were used.

On the day of injury (D1), the following surgery at the level II medical care (Role II) was performed: laparotomy, suturing of the right diaphragmal dome, suturing and tamponade of the liver with the portal blood flow exclusion for up to 15 min, right-sided nephrectomy, obstructive right-sided hemicolectomy, splenectomy, sanitation and drainage of abdominal cavity and retroperitoneal space on the right, and drainage of the pleural cavity on the right.

On D2, after a comprehensive examination the following surgery at the level III medical care (Role III) was performed: relaparotomy, tampons removal from the abdominal cavity and ileotransverse anastomosis.

**Fig. 1:**
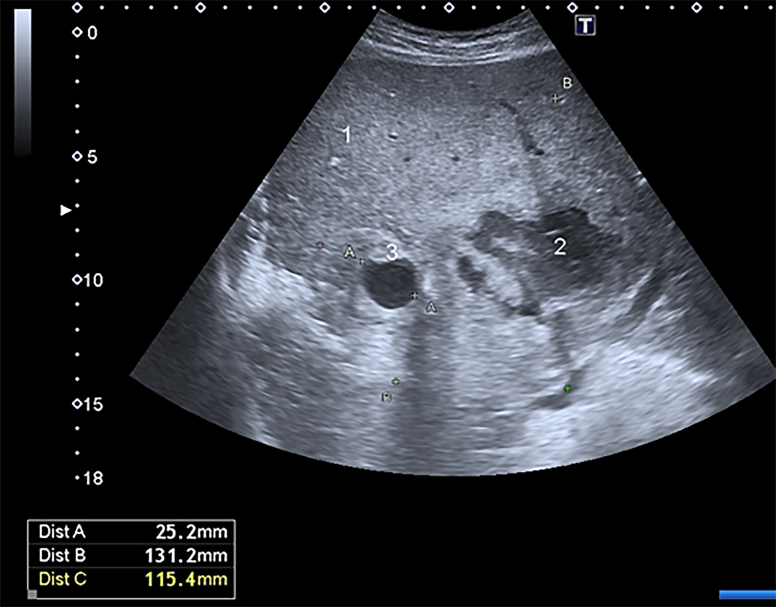
Convex probe, B-scanning mode. 1 — intact parenchyma, 2 — wound alteration area of liver, hematoma, 3 — pseudoaneurysm.

**Fig. 2:**
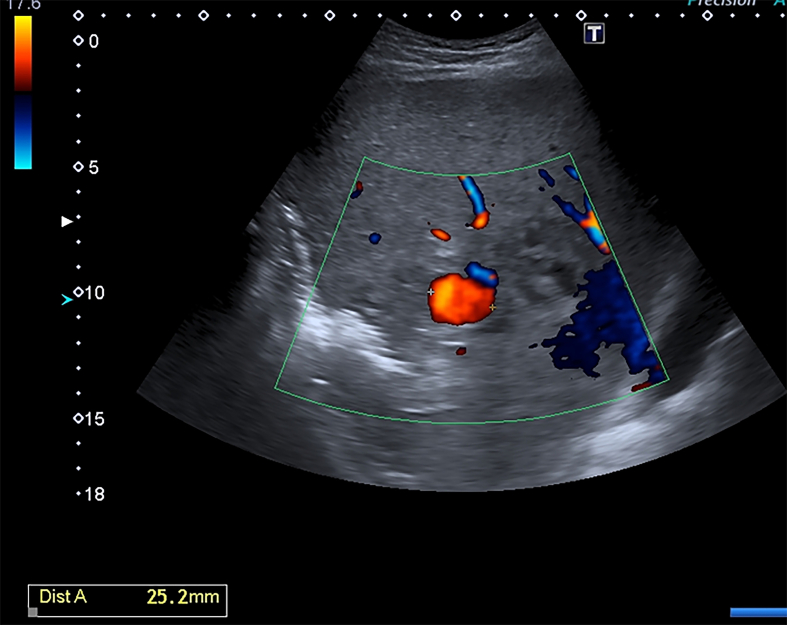
Convex probe. The right hepatic artery branch pseudoaneurysm in color mapping mode. https://media.giphy.com/media/v1.Y2lkPTc5MGI3NjExeXpqa3U5dGRxYWlxMG04bG51ZzZoN3UzcnVsOGNna2k1djN5NGRpeSZlcD12MV9pbnRlcm5hbF9naWZfYnlfaWQmY3Q9Zw/qqScGotE2ktK5YC32n/giphy.gif

On D5, there was evacuation at the level IV medical care (Role IV). During the examination, the patient's condition was serious, hemodynamics was stable. During the ultrasound observation with the use of an expert-class scanner an exudative process in the abdominal cavity was revealed. In the right pleural cavity there was an exudate with estimated volume up to 200 ml (VFPS formula) [7]. The area of alteration in the liver parenchyma (S5, S6, S7) (131·2 × 115·4 mm) was represented by a wound defect and necrotic changes of mixed echogenicity with excess gas. Also a hypoechoic hematoma in the form of clot and an echogenic area of surrounding parenchyma imbibition with blood in the wound channel with vascular pattern breaches were revealed. On the background of the changed parenchyma, in the projection of the right hepatic artery branch, a liquid formation of a rounded shape up to 25·2 mm in the largest dimension was found (Fig. 1). It had no capsule but artifacts of lateral shadows, and distal ultrasound enhancement, with as well as uneven adjacent parenchyma compaction were visualized. In the ZOOM mode, in the lumen of the formation the symptom of spontaneous contrast of blood with turbulent flow was determined. The presence of turbulent pulsatile blood flow was confirmed when scanning in the color mapping mode (Fig. 2).

The formation was identified as a pseudoaneurysm of the right hepatic artery branch. Its location corresponded to the right branch of the hepatic artery at the border of the branching of the S6-S7 segments. More accurate localization of the level of the aneurysm is complicated by alteration of the parenchyma and hematoma. Contrast angiography was not performed according to the patient's condition Previous ultrasound examinations did not reveal this complication. Previous ultrasound examinations did not reveal this complication.

On a spiral computed tomography (CT) without contrast at triage levels III and IV on D2–D4 the liver parenchyma structure lesion was detected and the pseudoaneurysm was not verified. The patient underwent multifactorial complex treatment with daily monitoring of pseudoaneurysm and positive dynamics was observed.

**Fig. 3:**
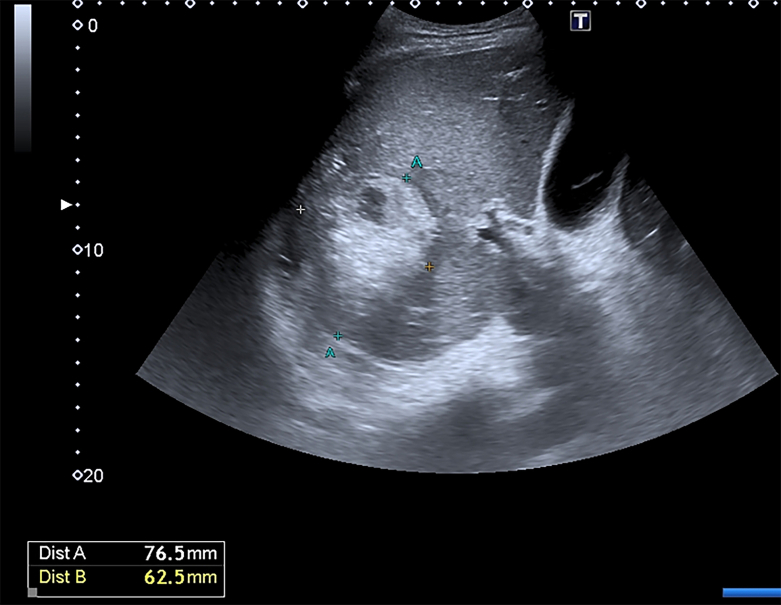
Convex probe, B-scanning mode. D23 after injury. Fibrous transformation of the liver damage area, thrombosis of pseudoaneurysm. https://media.giphy.com/media/v1.Y2lkPTc5MGI3NjExdGgyZDdwcGdvbWVoYzMxOGE1amNnNWp4Mm5xN2c0bGl4dGM4MzJmaCZlcD12MV9pbnRlcm5hbF9naWZfYnlfaWQmY3Q9Zw/rzjMFatTWpDzDOo4ni/giphy.gif

On D23, a control ultrasound examination was performed: no bile leakages were revealed, according to the visual assessment of drainage discharges and the absence of fluid accumulations around the liver during sonography; the locus of wound alteration was 76·5 × 62·5 mm and had signs of fibrous transformation, the hepatic artery pseudoaneurysm had significantly decreased in size, had a fixed clot in its lumen, and the blood flow was not determined by the color mapping (Fig. 3).

On D32, the stabilized patient was discharged for further rehabilitation.

### 2.2 Clinical case #2

On D1, а 35-year-old military serviceman suffered a severe blind gunshot thoracoabdominal wound with a right VIII rib fracture, through and through right liver lobe wound (S5, S6, S7, S8) (Moore IV) and right-sided post-traumatic pneumothorax. Medicines and medical devices regulated by the list of medications and devices prescribed for a certain pathology at a certain level of medical care were used.

On D1, the following surgery at the level II medical care (Role II) was performed: drainage of the right pleural cavity according to Bülau, laparotomy, stanching of the liver blood flow by suturing the damaged segments, tamponade of the suprahepatic and subhepatic spaces, sanitation and drainage of the abdominal cavity.

On D4, at the level III medical care (Role III) relaparotomy and extra tamponade of wounded liver sections were conducted.

On D7, relaparotomy, six gauze swabs removal and sanitation of the abdominal cavity were conducted.

**Fig. 4:**
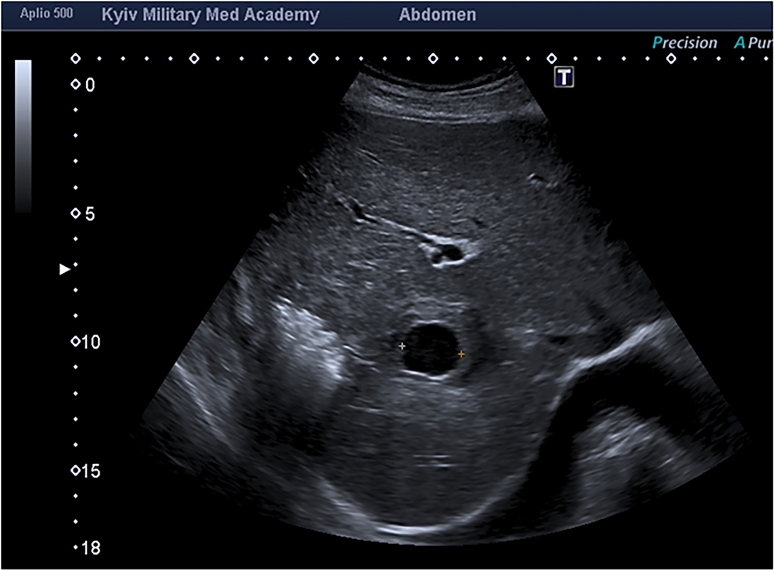
Convex probe, B-scanning mode. The right hepatic artery pseudoaneurysm size of 25·6 × 21·3 mm against a background of a massive area of posttraumatic right liver lobe alteration. https://media.giphy.com/media/v1.Y2lkPTc5MGI3NjExcGpiejltMDQzZHJhenR2cGFqaDN5YW84NHo0dTgzNzE4Y2J5aDNkaSZlcD12MV9pbnRlcm5hbF9naWZfYnlfaWQmY3Q9Zw/PdVwTWGE3AZoTBCRnp/giphy.gif

On D9, there was an evacuation at the level IV medical care (Role IV). The patient's condition was severe, hemodynamiсs was stable. An ultrasound examination with an expert-class scanner showed a large right liver lobe lesion in the form of parenchymal imbibition with blood, with destruction loci filled with excess gas. A limited fluid formation of 25·6 × 21·3 mm with an artifact of distal ultrasound enhancement was visualized in the projection of the right hepatic artery branch, it had no capsule, contained an echogenic fluid, and the adjacent parenchyma of the liver was unevenly compacted (Fig. 4).

In the zoom mode a symptom of spontaneous blood contrast was observed. In the mode of color Doppler mapping, a pulsating blood flow was registered. Thе pulsating cavity was identified as a pseudoaneurysm of the right hepatic artery branch. Its location was corresponded to the right branch of the hepatic artery distal to the S8 branch. More accurate localization of the level of the aneurysm is complicated by alteration of the parenchyma and hematoma. Contrast angiography was not performed according to the patient's condition.

On D4 and D9, with a spiral CT without contrast at the level III and IV medical care in a triage course a wound channel was detected, and liver parenchyma structure lesion was observed, but pseudoaneurysm was not verified. The patient underwent multifactorial complex treatment.

**Fig. 5:**
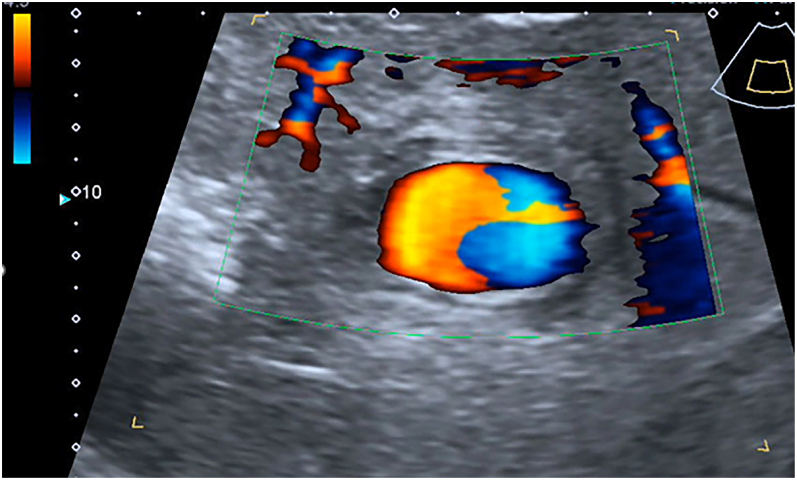
Convex probe, Duplex color scanning mode, zoom mode, a picture from the monitor screen in the intensive care unit. Turbulent blood flow in the pseudoaneurysm cavity.

**Fig. 5.1:**
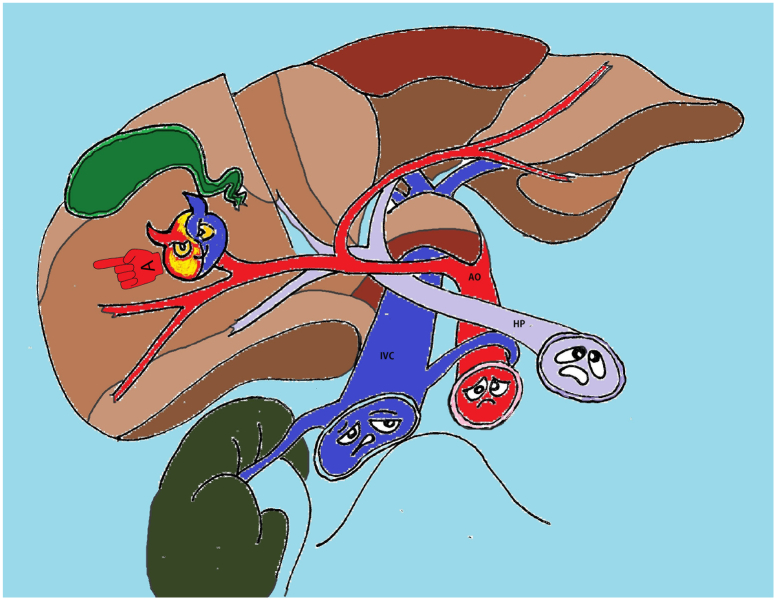
AO – aorta, IVC – inferior vena cava, HP – hepatic portal vien, A – pseudoaneurysm. https://media.giphy.com/media/v1.Y2lkPTc5MGI3NjExcGpiejltMDQzZHJhenR2cGFqaDN5YW84NHo0dTgzNzE4Y2J5aDNkaSZlcD12MV9pbnRlcm5hbF9naWZfYnlfaWQmY3Q9Zw/PdVwTWGE3AZoTBCRnp/giphy.gif

On D11, blood began to be released from subphrenic space by drains, the blood pressure decreased to 80/60 mmHg, and Hb obtained was 82 g/l. An emergency ultrasound examination was performed in the intensive care unit. The free liquid in the estimated volume of more than 500 ml in the form of clots and hemolyzed blood in the abdominal cavity was detected. The pseudoaneurysm increased to 31·8 mm in the largest dimension (Fig. 5**,** a picture from the monitor screen in the intensive care unit) (Fig. 5.1).

**Fig. 6:**
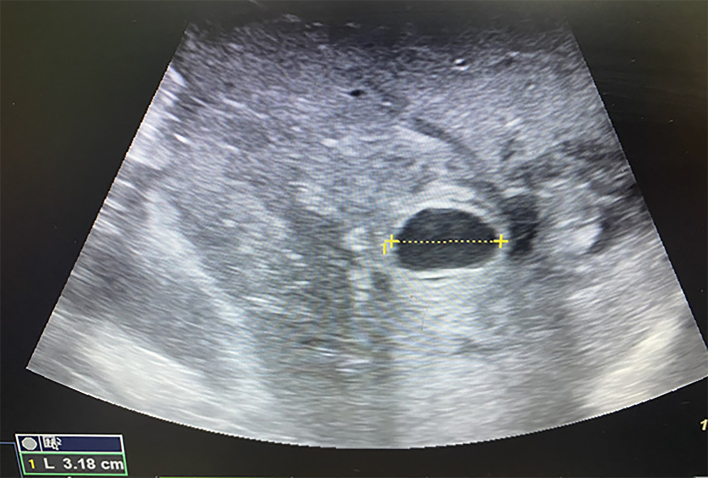
Convex probe, B-scanning mode, a picture from the monitor screen in the intensive care unit.

**Fig. 7:**
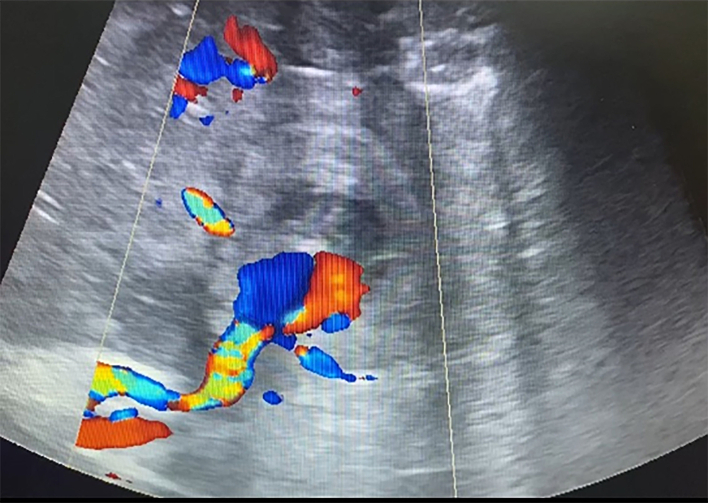
Convex probe, Duplex color scanning mode, a picture from the monitor screen in the operating theatre. Deformity of the pseudoaneurysm as a result of its rupture with a change in the parenchyma's texture due to blood imbibition. https://media.giphy.com/media/v1.Y2lkPTc5MGI3NjExZ3ZmdGlwYzF3bWY1OWQyZXRob3J3b204ZHlldDBic2czN3ozZTVnaCZlcD12MV9pbnRlcm5hbF9naWZfYnlfaWQmY3Q9Zw/L20Nz6o0Dwc1xBl3zP/giphy.gif

Conservative measures proved to be ineffective, the bleeding continued. On ultrasound examination in the operating theatre the imbibition of the parenchyma with blood increased, with the fluid accumulation under the diaphragm (Fig. 6, Fig. 7).

Enlargement of the hepatic artery pseudoaneurysm to 31·8 mm.

https://media.giphy.com/media/v1.Y2lkPTc5MGI3NjExbHRmM3hwd2E3dXUxenlmajIzbDh3NHJham1ndTZ0a2ZpODg0aGExNCZlcD12MV9pbnRlcm5hbF9naWZfYnlfaWQmY3Q9Zw/6uoGJIZWpQEZaMKFbB/giphy.gif

On D11 + 2 h, an emergency relaparotomy was performed with repeated extra tamponade of the liver lesions and subphrenic space. Hemostasis was achieved. Considering the size of aneurysm and the risk of repeated bleeding, on D12, the patient underwent endovascular embolization of the right hepatic artery branch pseudoaneurysm. An occlusion was achieved.

On D66, the stabilized patient was discharged for further rehabilitation.

## 3 Discussion

Liver damage is one of the most common consequences of abdominal injuries and wounds, the management of which requires a multidisciplinary approach using modern methods of diagnostics and treatment. According to the data in literature, one third of all cases are severe (WSES IV–V, Moor III–IV), associated with bile ducts and vessels, massive bleeding and bile leakage. One of the rare complications is the hepatic artery pseudoaneurysm, which occurs in 0·4 % of cases and carries the risk of further rupture and a threat to a patient's life [4]. According to our own experience, we observed the occurrence of pseudoaneurysms in 3·2 % of patients with liver injuries. In the modern literature the list of sources covering ultrasound diagnostics, monitoring and consequences of arterial pseudoaneurysm treatment in liver injuries is extremely limited.

Clinical cases presented here belong to the category of severe injuries caused by high-energy weapons, which are characterized by a syndrome of mutual aggravation and need for simultaneous treatment of several damaged organs. In both cases, the method of staged surgical treatment was applied, in accordance with the circumstances of wartime. Liver injuries were accompanied by massive bleeding and bile leakage, had massive areas of necrotic changes, and microbial contamination was detected. Under such conditions, the course of post-traumatic period normally becomes potentially dangerous in the context of complications and requires constant clinical, laboratory and instrumental monitoring.

In the first case above, on the background of complex treatment on D23 from the injury, we observed thrombosis of the pseudoaneurysm, and in the second case on D11 there was the rupture of the pseudocapsule and internal bleeding. Unfavorable prognostic ultrasound criteria were: an increase in size of the pulsating cavity, its deformity, change in the echostructure of adjacent parenchyma in a short period of time and the appearance of fluid accumulations in the liver parenchyma and subphrenic space. In our case, a clear correlation between the aneurysm rupture and the deterioration of the patient's general condition was observed, manifested by the release of blood through the drains, as well as the occurrence of acute anemia and hemodynamic disorders (pressure drop).

Ultrasound examination with the use of color Doppler program helped to reveal the process of the spontaneous thrombosis and rupture of pseudoaneurysm with signs of internal bleeding, taking minimal loss of time and technical capabilities.

The results of these two clinical observations are not reliable evidence, but they suggest that a spiral computed tomography without contrast is incapable to visualize a pseudoaneurysm on the background of massive liver hemobilomas - in our cases the examination was performed twice by different specialists, with the use of different tomographs and with different intervals after the injury was obtained. The use of contrast methods in severely injured patients requires instrumental justification, and results of daily ultrasound monitoring with the use of color Doppler program can be the one.

To date, many questions remain unanswered regarding the occurrence, prognosis and effectiveness of pseudoaneurysm treatment in liver injuries, thus further research remains relevant.

## 4 Conclusions

With severe liver damage as a result of trauma or injury, pseudoaneurysms of branches of the hepatic artery were observed in 3.2 % of victims with cases of both spontaneous thrombosis and rupture with massive bleeding, which required endovascular embolization.

For medical sorting and monitoring of such patients, it is necessary to involve highly qualified sonographers with expert-class scanners.

Evacuation of patients at III and IV levels of medical care (similar to Role III-IV) should be directed to medical facilities equipped with endovascular correction technologies.

## Disclaimer

Consent was obtained from patients to publish these case reports, including given information and sonographic images. No competing interests to be declared. No conflict of interest to be reported. We received no financial support in publishing this case or any steps of preparation. No AI function was used. These cases were not presented at a conference or regional meeting up to date.

These case reports were reported in line with PROCESS [19] criteria and SCARE checklist [20].

## Consent

Written informed consent was obtained from the patient for publication of this case report and accompanying images. A copy of the written consent is available for review on request.

## Ethical approval

Written informed consent was obtained from the patient for publication of this case report and accompanying images. A copy of the written consent is available for review on request.

Ethical approval - Ukrainian Military Medical Academy, Kyiv, Ethical Committee.

## Funding

The authors declare that they had no sponsorship.

## Author contribution

Svitlychnyi E.V.: conceptualization, data curation, observation and investigation, supervision, writing original draft.

Kochmaruk O.A.: conceptualization, visualization, review, editing and translation, assistance in literature search and review.

## Guarantor

Svitlychnyi E.V.

## Research registration number

Not applicable.

## Conflict of interest statement

The authors declare that they have no conflict of interest.
